# Melanotic Neuroectodermal Tumor of Infancy of Jaws: A Case Report and Review of Literature

**DOI:** 10.7759/cureus.57116

**Published:** 2024-03-28

**Authors:** Meleti V Sowmya, Uma Shanker Pal, Mala Sagar, Ranjeet Singh

**Affiliations:** 1 Department of Oral and Maxillofacial Surgery, Sarojini Naidu Medical College, Agra, IND; 2 Department of Oral and Maxillofacial Surgery, King George's Medical University, Lucknow, IND; 3 Department of Pathology, King George's Medical University, Lucknow, IND

**Keywords:** recurrence local, anterior maxilla, pigmented tumor, pediatric jaw tumor, melanotic neuroectodermal tumor of infancy

## Abstract

Melanotic neuroectodermal tumor of infancy (MNTI) is an uncommon pigmented neural crest tumor primarily found in infants. We presented a case report of successful surgical management of MNTI in an 11-month-old female. A total of 178 articles discussing 249 cases of MNTI were identified through literature search. Literature review of 250 cases of MNTI including the current case report was conducted considering study parameters such as age and gender of the patient, location of the lesion, levels of vanillylmandelic acid, management options, and outcome after treatment. Statistical review of the data showed that MNTI predominantly affects the anterior maxilla in infants less than six months of age. Recurrence of the lesion shows a significant association with age of the individual and treatment method employed. This study reports a 2.4% mortality rate, 2% malignancy rate, and a recurrence rate of 15.2%, with recurrence times ranging from 15 days to 20 months. We advocate a minimum follow-up of four months to two years to monitor recurrence.

## Introduction

Melanotic neuroectodermal tumor of infancy (MNTI) is a benign, pigmented tumor of neural crest origin. It is a rare lesion. A recent systematic review recorded 371 cases of MNTI in jaw bones in the literature since its first description by Krompecher in 1918 [1]. There are up to 14 confusing terminologies used previously to address this tumor, but Borello and Gorlin in 1966 confirmed the neural crest origin of this tumor, naming it the “melanotic neuroectodermal tumor of infancy” [2]. The majority of cases are reported in the infantile age group, and the maxilla is the commonest site for this lesion. Even though a rare lesion, thorough knowledge regarding MNTI is much needed for an oral surgeon, as this lesion can mimic an odontogenic lesion and is predominant in infants. Improper diagnosis and management lead to permanent facial disfigurement, lifetime morbidity, and rarely death. This article adds one more case of MNTI managed successfully by surgery and presents a review of the literature regarding MNTI, compiling and interpreting the data available on MNTI in jaw bones.

## Case presentation

An 11-month-old female child presented to our departmental outpatient unit with a chief complaint of swelling over the right side of the upper jaw. The child's parents noticed the growth when she was five months old.

**Figure 1:**
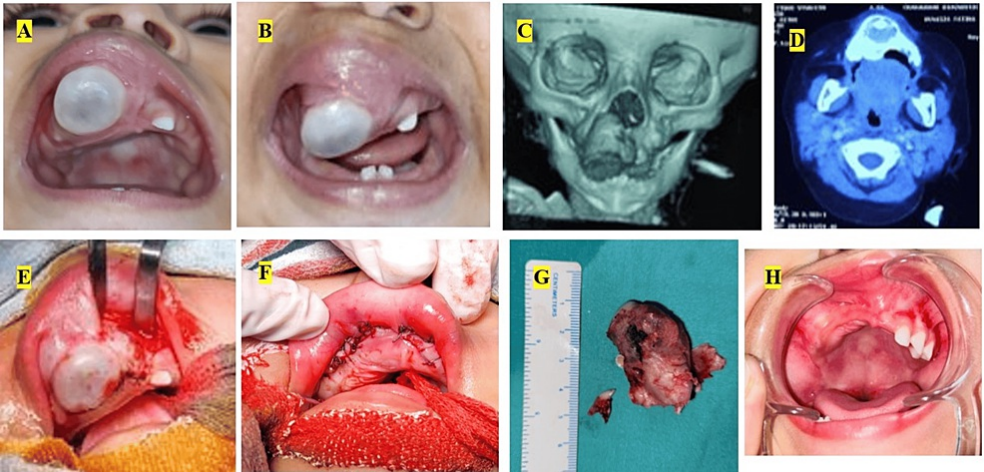
(A) Intraoral image showing buccopalatal extension of the tumor. (B) Intraoral image showing superoinferior extension of the tumor. (C) 3D reconstructed computed tomographic image showing the tumor. (D) Axial cut of computed tomographic image showing the tumor. (E) Intraoperative picture of tumor enucleation. (F) Intraoperative picture after primary closure of the surgical defect. (G) Surgical specimen. (H) Clinical image during two years’ follow-up with no recurrence.

She was the only child of healthy parents in a non-consanguineous marriage. Upon eliciting history, the child's parents revealed that the child was healthy at the time of birth. The swelling was rapidly progressive, causing difficulty in feeding. The swelling was not associated with any episodes of remission or discharge of fluids. There was no familial history of such swelling. Upon examination, the lesion caused disfigurement of the face with swelling protruding inferiorly beyond the limit of the upper lip vermilion and pushing the right ala of the nose superiorly. Intraorally, the swelling was 2x2 cm in dimension, extending anteroposteriorly, involving the right anterior maxilla, and buccopalatally, extending from the buccal vestibule to the palatal gingiva across the alveolar ridge. The swelling had the appearance of a coin-sized marble-like elevation (blanching of overlying mucosa) with a central melanaceous hue. Upon palpation, the swelling was non-tender, firm in consistency, and non-compressible (Figures 1A, 1B).

Considering the age, site, and melanaceous hue, a provisional diagnosis of MNTI was made. Computed tomography was advised, which revealed a radiolucent osteodestructive lesion involving the right anterior maxilla with definite borders, sparing the maxillary sinus, and pushing the nasal floor superiorly. A radiopaque tooth-like structure can be seen within the radiolucent interior of the lesion (Figures 1C, 1D).

After obtaining informed consent, excisional biopsy was planned under general anesthesia. The patient was orally intubated. The lesion was approached through an intraoral vestibular incision. A cleavage plane, between the lesion and adjacent bone, led to the in-toto excision of the lesion (Figure 1E). The adjacent bone was clinically normal. Primary closure of the defect was performed (Figure 1F). Postoperative healing was uneventful.

Gross examination of the surgical specimen revealed areas of dark pigmentation (Figure 1G). The cut surface showed areas of grayish-white to grayish-brown areas.

**Figure 2:**
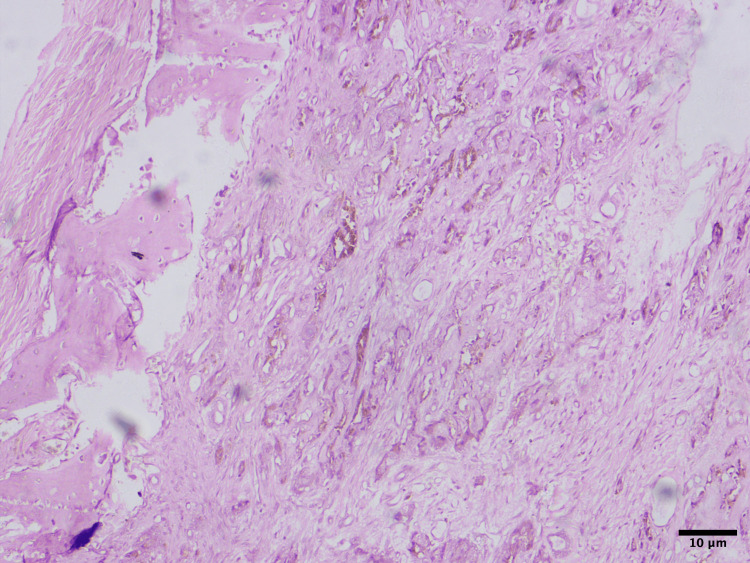
Histopathologic image showing areas of pigmentation. Hematoxylin and eosin staining (x200 magnification)

Histopathological slides showed areas of small round to oval neuroblast-like cells and large melanin-producing epithelioid cells arranged in alveolar pattern showing cords and trabeculae with bony trabeculae intersected by fibrous stroma (Figure 2).

**Figure 3:**
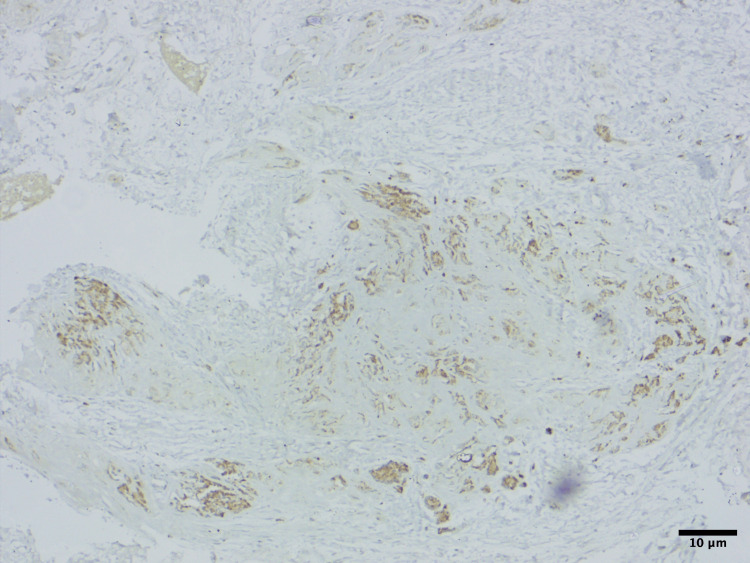
Immunohistochemistry showing the epithelioid cells to be positive for HMB45 (x100 magnification).

**Figure 4:**
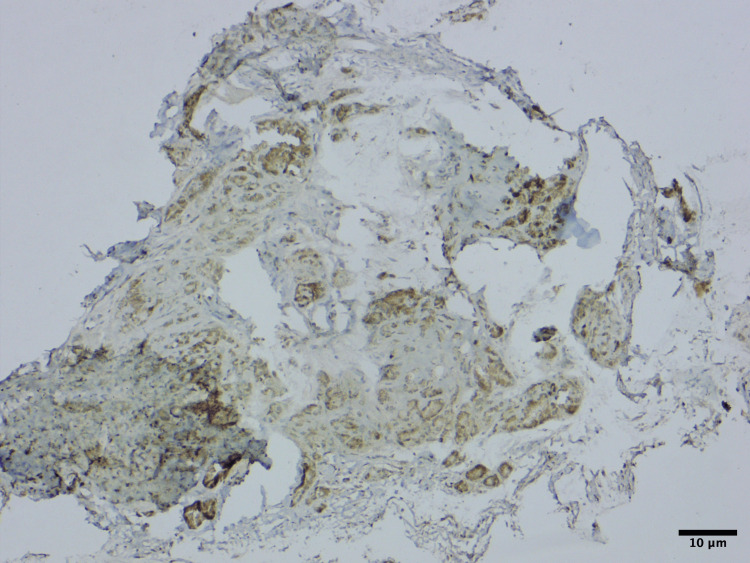
Immunohistochemistry showing both small and large tumor cells to be positive for synaptophysin (x100 magnification).

**Figure 5:**
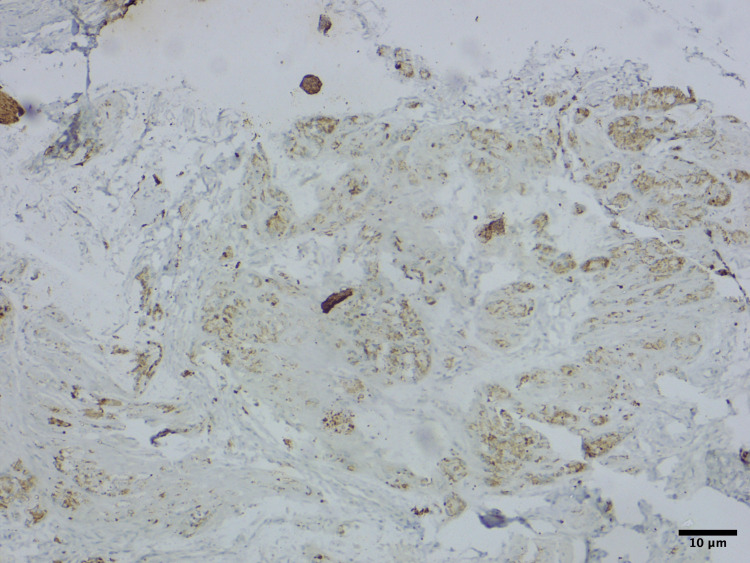
Immunohistochemistry showing the epithelioid cells to be positive for cytokeratin (x100 magnification)

Immunohistochemistry proved the tissue to be positive for HMB-45 (Figure 3), synaptophysin (Figure 4), and cytokeratin (CK) (Figure 5). These findings are consistent with the diagnosis of MNTI.

One-month follow-up showed no signs of any recurrence. Regular follow-up was conducted for two years, and the patient is still symptom-free (Figure 1H).

## Discussion

Search strategy

An electronic search was conducted in search engines such as PubMed, Google Scholar, and ScienceDirect using the terminology "melanotic neuroectodermal tumor of infancy." Abstracts of all those articles obtained through the search were screened to get articles related to "melanotic neuroectodermal tumor of infancy of jawbone." Reference articles of these selected articles were also screened to get more relevant articles. Hand search in a few related journals was also conducted. The final selection of the articles was based on the inclusion and exclusion criteria.

Inclusion criteria

Publications reporting the cases of histologically proven MNTI of jawbones were included in the study.

Exclusion criteria

The study excluded publications in which they mentioned the final diagnosis with previously used terminologies of MNTI, such as melanotic progonoma, retinal anlage tumor, and pigmented ameloblastoma, to avoid any misinformation because of confusion in these terminologies leading to wrong diagnosis. Studies reporting MNTI affecting areas other than jawbones were also excluded.

Study selection and data extraction

**Figure 6:**
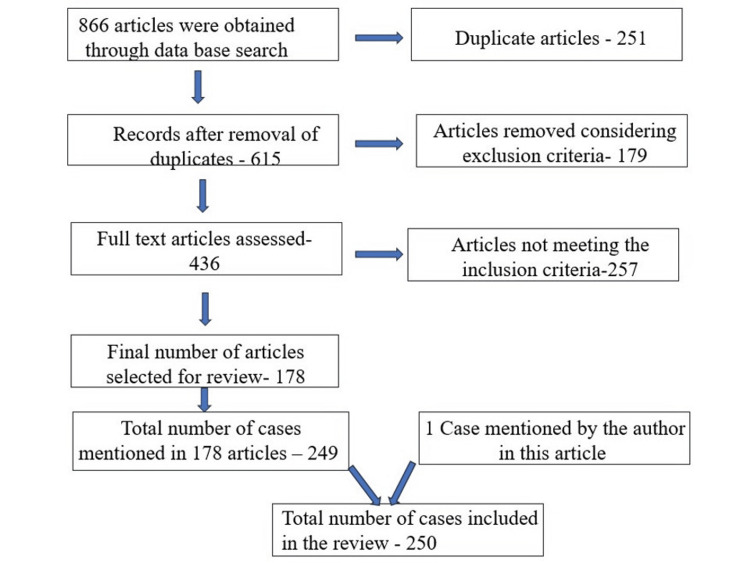
Flow chart showing the selection of articles based on inclusion and exclusion criteria.

Primary screening was conducted by two authors independently by screening the titles and abstracts of all the potential publications obtained through the search. After removing the duplicate articles and articles not following the inclusion criteria, full-text screening of the remaining articles was conducted to record the parameters such as age, sex, location, management option, recurrence, and urine VMA levels. Any disagreement among the authors was solved by discussion. A total of 178 articles discussing 249 cases of MNTI in the jawbone were selected for data extraction. Schema of the article selection is depicted in the flow chart (Figure 6).

Data extraction was conducted by two authors independently. From the 178 articles, the parameters were recorded in an Excel sheet and analyzed. The analysis included age distribution, gender distribution, clinical presentation, radiological features, histopathological characteristics, treatment modalities, recurrence rates, outcomes, and factors influencing recurrence. The final number of cases included in this review was 250 including the current case we described.

Results

Site distribution of the lesion shows that 101 (40.4%) cases were associated with the anterior region, four (1.6%) cases were related to the posterior region, and for 145 (58.0%) cases site was not mentioned, highlighting the prevalence of cases in the anterior region and a smaller representation in the posterior region.

The distribution of cases based on location revealed that most of the cases were associated with the maxilla, constituting 233 cases, which accounts for 93.2% of the total. In contrast, the mandible had a lesser representation, with 17 cases, making up 6.8% of the total cases. This distribution provides a clear perspective on the prevalence of cases in the maxilla compared to the mandible.

**Figure 7:**
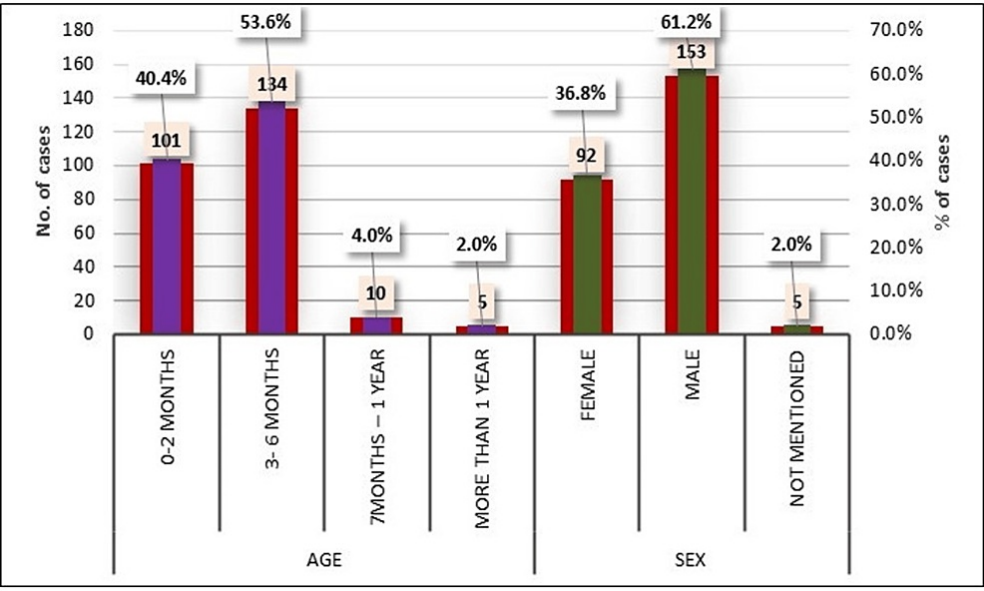
Distribution of cases based on age and sex.

 In terms of age, most cases were observed in the age group of three to six months, accounting for 134 (53.6%) cases, followed by zero to two months, accounting for 101 (40.4%) cases, while the categories of seven months to one year and more than one year represented 10 cases (4.0%) and five (2.0%) cases, respectively. Regarding gender distribution, females comprised 92 (36.8%) cases, males constituted a larger portion with 153 (61.2%) cases, and there were five cases (2.0%) where the gender was not specified (Figure 7).

**Figure 8:**
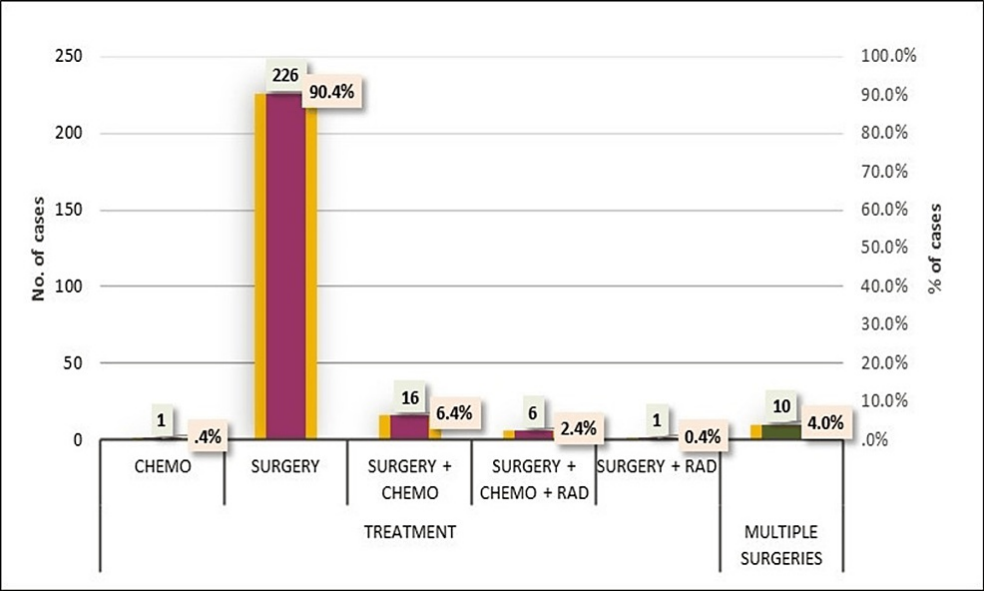
Distribution of cases according to treatment.

The treatment profile of the patients revealed a predominant reliance on surgical interventions, with 226 (90.4%) cases undergoing surgery as the primary treatment modality. Further nuances in treatment strategies were observed, with 16 (6.4%) cases opting for a combination of surgery and chemotherapy, and six (2.4%) cases receiving a comprehensive approach involving surgery, chemotherapy, and radiation therapy. Additionally, one (0.4%) cases underwent surgery combined with radiation therapy, while one (0.4%) case involved chemotherapy alone (Figure 8). In a smaller subset of cases, comprising 10 (4.0%) cases, patients had undergone multiple surgical interventions.

**Figure 9:**
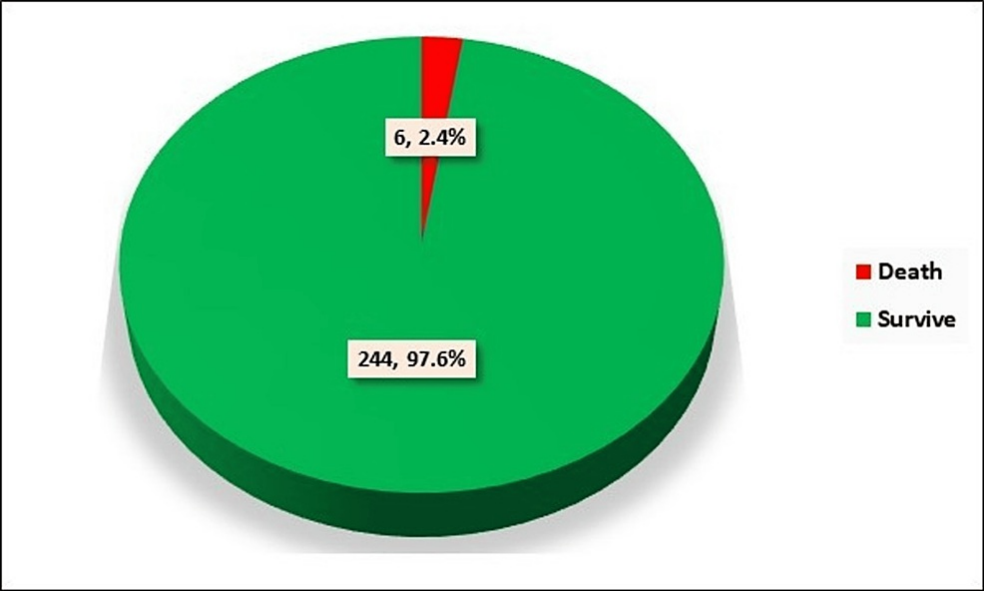
Distribution of cases according to outcome. Numerical values indicate the number of cases and percentage of cases, respectively.

The outcomes of the patients under consideration revealed a notably high survival rate, with 244 (97.6%) cases successfully overcoming the challenges associated with the disease. Unfortunately, there were six (2.4%) cases where the outcome was less favorable, resulting in the death of the patient (Figure 9).

**Figure 10:**
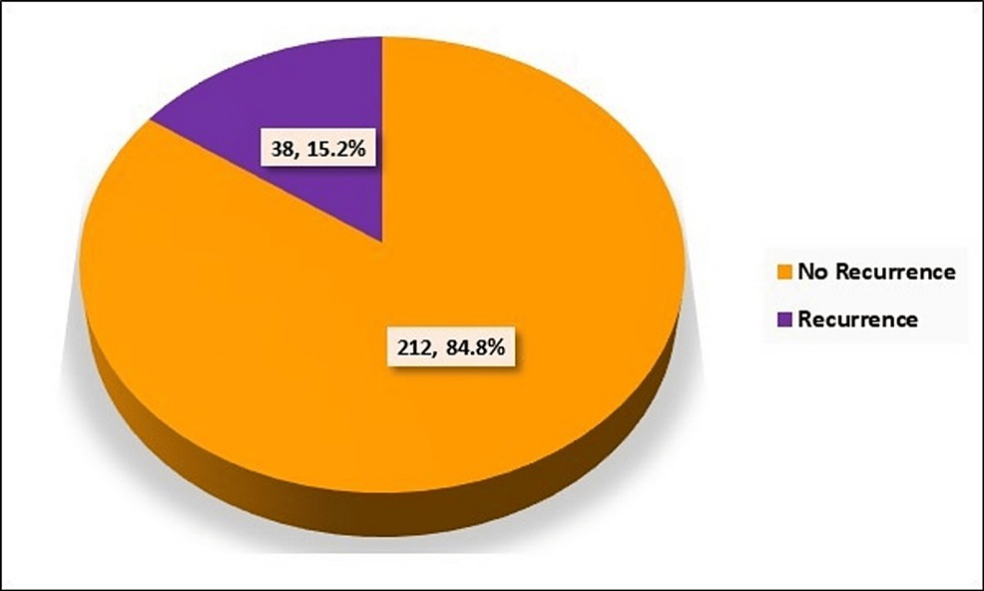
Distribution of cases according to recurrence. Numerical values indicate the number of cases and percentage of cases, respectively.

The data regarding recurrence in the patient cohort indicated a predominant trend of non-recurrence, with 212 (84.8%) cases showing no signs of the disease recurrence. However, there were 38 (15.2%) cases where recurrence was observed (Figure 10).

But these values may not show the actual condition as it is a retrospective study, and in most of the case reports, the follow-up period was less and recurrence might have occurred at later dates.

Data on vanillylmandelic acid (VMA) levels in the patient population revealed that 31 (12.4%) cases exhibited normal levels, while 22 (8.8%) cases indicated elevated VMA levels. In 197 (78.8%) cases, lacked information regarding VMA levels.

The data on malignancy status revealed that most cases, constituting 245 cases (98.0%), did not exhibit malignancy. In contrast, a small proportion of cases, specifically five cases (2.0%), were identified as having malignancy. This distribution emphasizes the predominantly non-malignant nature of the cases under consideration, with only a minority presenting malignancies (Table 1).

**Table 1:** Distribution of cases according to malignancy.

Malignancy	No.	%
No	245	98%
Yes	5	2%

The distribution of recurrence across different anatomical locations, specifically the mandible and maxilla, reveals noteworthy patterns. In the mandibular cases, 13 (76.5%) cases experienced no recurrence, while four (23.5%) cases exhibited recurrence. This suggests a relatively balanced recurrence profile in mandibular cases, with no statistically significant difference noted (chi-square = 0.98, p = 0.322).

In contrast, maxillary cases demonstrated a higher recurrence rate, with 199 (85.4%) cases showing no recurrence and 34 (14.6%) cases experiencing recurrence. Although a numerical difference exists, the chi-square analysis indicates that this difference is not statistically significant (Table 2).

**Table 2:** Association of recurrence with location. Chi-square test

Location	No recurrence		Recurrence		Significance
	No.	%	No.	%	
Mandible	13	76.5%	4	23.5%	chi square=0.98, p=0.322
Maxilla	199	85.4%	34	14.6%	

The analysis of recurrence patterns based on age groups reveals that in the age category of zero to two months, there were 76 (75.2%) cases without recurrence and 25 (24.8%) cases with recurrence. This age group showed a statistically significant association with recurrence (chi-square = 12.98, p = 0.005), indicating that infants aged zero to months are more likely to experience recurrence compared to other age groups.

For the age group of three to six months, the majority of cases (121 cases, 90.3%) did not exhibit recurrence, while 13 (9.7%) cases did. No recurrence was observed in the seven months to one year and more than one-year age categories, suggesting a higher likelihood of recurrence in the younger age group (Table 3). These findings emphasize the importance of considering age as a potential risk factor.

**Table 3:** Association of recurrence with age. Chi-square test

Age	No recurrence		Recurrence		Significance
	No.	%	No.	%	
0-2 months	76	75.2%	25	24.8%	Chi square=12.98, p=0.005
3-6 months	121	90.3%	13	9.7%	
7 months to 1 year	10	100%	0	0%	
More than 1 year	5	100%	0	0%	

The analysis of recurrence patterns based on gender indicates that there is no statistically significant association between sex and recurrence. In the female group, 78 (84.8%) cases did not experience recurrence, while 14 (15.2%) cases did. Similarly, in the male group, the majority of cases (129 cases, 84.3%) did not exhibit recurrence, and 24 (15.7%) cases did. The chi-square test yielded a p-value of 0.630, suggesting that there is no significant difference in recurrence rates between males and females (Table 4).

**Table 4:** Association of recurrence with sex. Chi-square test

Sex	No recurrence		Recurrence		Significance
	No.	%	No.	%	
Female	78	84.8%	14	15.2%	Chi square=0.92, p=0.630
Male	129	84.3%	24	15.7%	
Not mentioned	5	100%	0	0%	

The analysis of recurrence in relation to treatment modalities revealed significant associations between the type of treatment and recurrence rates. Surgical interventions, either alone or in combination with chemotherapy and/or radiation, exhibited varying recurrence rates.

Among cases treated solely with surgery, 198 (87.6%) cases experienced no recurrence, while 28 (12.4%) cases did. For cases treated with surgery and chemotherapy, 10 (62.5%) cases did not experience recurrence, whereas 6 (37.5%) cases did. The combination of surgery and chemotherapy with radiation resulted in two (33.3%) cases without recurrence and four (66.7%) cases with recurrence. A single case treated with surgery and radiation showed no recurrence. One case that received chemotherapy (chemo) as a standalone treatment showed no recurrence.

The chi-square test revealed a highly significant association between the type of treatment and recurrence (p<0.001) (Table 5). This underscores the crucial role of treatment strategies in influencing the recurrence.

**Table 5:** Association of recurrence with treatment. Chi-square test

Treatment	No recurrence		Recurrence		Significance
	No.	%	No.	%	
Chemotherapy	1	100.0%	0	0.0%	Chi square=20.25, p<0.001
Surgery	198	87.6%	28	12.4%	
Surgery + chemotherapy	10	62.5%	6	37.5%	
Surgery + chemotherapy + radiotherapy	2	33.3%	4	66.7%	
Surgery + radiotherapy	1	100.0%	0	0.0%	

The analysis of VMA levels in relation to recurrence rates reveals that among cases with normal VMA levels, 23 (74.2%) cases experienced no recurrence, while eight (25.8%) cases had a recurrence. In instances where VMA levels were raised, 20 (90.9%) cases had no recurrence, and only two (9.1%) cases had a recurrence.

Although the chi-square test did not yield a statistically significant association between VMA levels and recurrence (p=0.175), the trend suggests potential implications (Table 6).

**Table 6:** Association of recurrence with VMA level. Chi-square test VMA, vanillylmandelic acid

VMA level	No recurrence		Recurrence		Significance
	No.	%	No.	%	
Normal	23	74.2%	8	25.8%	Chi square=3.49, p=0.175
Not mentioned	169	85.8%	28	14.2%	
Raised	20	90.9%	2	9.1%	

The association between the presence of malignancy and the recurrence shows that among cases without malignancy, 211 (86.1%) cases showed no recurrence, while 34 (13.9%) cases experienced a recurrence. In contrast, among cases with malignancy, only one (20.0%) cases showed no recurrence, and four (80.0%) cases had a recurrence.

The chi-square test revealed a highly significant association between the presence of malignancy and recurrence (p<0.001) (Table 7). This underscores the importance of malignancy status as a critical factor influencing the likelihood of recurrence in MNTI. The findings emphasize the need for comprehensive management strategies for cases with malignancy to reduce the risk of recurrence and improve patient outcomes.

**Table 7:** Association of recurrence with malignancy. Chi-sqaure test

Malignancy	No recurrence		Recurrence		Significance
	No.	%	No.	%	
No	211	86.1%	34	13.9%	Chi square=16.62, p<0.001
Yes	1	20.0%	4	80.0%	

The descriptive summary of recurrence time in months among different treatment modalities for MNTI shows that the mean recurrence time for cases treated with surgery alone is 2.44 months, with a standard deviation of 3.66 months, ranging from 15 days to 20 months. Cases treated with a combination of surgery and chemotherapy have a lower mean recurrence time of 1.65 months, demonstrating less variability with a standard deviation of 1.24 months and a range of 15 days to 3.00 months. On the other hand, cases treated with surgery, chemotherapy, and radiation collectively exhibited a significantly higher mean recurrence time of 13.33 months, accompanied by a standard deviation of 8.08 months and a range of 4 to 18 months.

The overall mean recurrence time across all treatment modalities is 3.26 months, with a standard deviation of 4.92 months and a range of 15 days to 20.00 months. The analysis of variance (ANOVA) indicates a statistically significant difference in recurrence time among the treatment groups (F=11.06, p<0.001) (Table 8). This suggests that the choice of treatment modality significantly influences the time to recurrence in MNTI.

**Table 8:** Descriptive summary of recurrence time and comparison with treatment modalities. ANOVA test ANOVA, analysis of variance

Treatment	Recurrence in months			
	Mean	SD	Min	Max
Surgery	2.44	3.66	0.50	20.00
Surgery + chemotherapy	1.65	1.24	0.50	3.00
surgery + chemotherapy + radiotherapy	13.33	8.08	4.00	18.00
Total	3.26	4.92	0.50	20.00
ANOVA	F=11.06, p<0.001			

Discussion

MNTI, as the name describes, is a pigmented tumor of neural crest origin usually seen in infants. There are three cases reported in adults, with the maximum age of presentation being 41 years [3]. Fifteen cases of MNTI were identified at the time of birth. Koob et al. in 2015 reported a case of in utero diagnosis of orbital MNTI using fetal ultrasound and fetal magnetic resonance imaging (MRI) [4]. The maxilla is the most common site of occurrence of MNTI in the craniofacial area, followed by the skull, mandible, orbit, and zygoma [5]. Soft tissue MNTI of the cheek has also been reported [6]. MNTI shows a male predilection. There are several theories explaining the histogenesis of MNTI, of which the neural crest theory is most accepted [7]. Histochemical and ultrastructural findings confirmed the neural crest origin of cells of MNTI [8].

MNTI has a rapid course of growth, and lesions up to 18 cm are also reported in the literature [9]. Depending on the size of the swelling, symptoms can range from facial asymmetry, difficulty in feeding, to epistaxis. Due to the melanin content of the tumor, clinically, the lesion will appear as a bluish-black swelling usually draped by the oral mucosa. Maxillary swelling with a bluish-black hue and a rapid course in an infant can be considered as a clinical pathognomic sign to diagnose an oral MNTI.

Radiographically, a well-defined expansile mass in the infantile maxilla or mandible can be an imaging characteristic supporting a radiologic interpretation of MNTI [10]. The effect on the surrounding bone can be reactive osteosclerosis or indentation or destruction of bone giving a sunburst or spiculated appearance on computed tomography. Internal calcification can be seen with or without an embedded tooth. Maxillary MNTI can cause sinonasal destruction and, at times, orbital extension. Contrast-enhanced computed tomography can help in the proper delineation of the soft tissue margins. On MRI, it appears as an isointense-hypointense lesion on T1-weighted images and hypointense on T2-weighted images. Melanin has a paramagnetic effect that causes a hyperintense signal on T1-weighted images in the case of densely pigmented lesions [11].

Macroscopically, the lesion is encapsulated with a fibrous osseous capsule, and the cut surface shows grayish-black discoloration because of melanin content. The hallmark microscopic feature of MNTI is the presence of a biphasic population of cells consisting of larger epithelioid melanogenic cells and smaller primitive neuroblast-like cells dispersed in dense fibrocollagenous stroma. The neuroblast-like component is regarded as the aggressive part of the neoplasm, and if neuroblasts are positive for Ki-67 or CD99, it appears to be associated with an aggressive type of MNTI [12]. The differential diagnosis of MNTI includes small round blue cell tumors such as neuroblastoma, rhabdomyosarcoma, lymphoma, and Ewing's sarcoma [13]. Immunohistochemical studies show that cytokeratin and HMB-45 (melanocyte-specific antibody) are positive in the epithelioid cells and that synaptophysin is positive in the neuroblast-like cells [12]. Both cell types show positivity for neuron-specific enolase and vimentin. Fine needle aspiration cytology, along with immunohistochemistry, helps as an excellent preoperative tool in differentiating MNTI from other lesions with similar histology. Immunohistochemistry was necessary to confirm the diagnosis in 45.9% of the patients [1].

Raised VMA levels can indicate a neuroectodermal tumor, but its absence does not rule out the disease. According to our study, VMA levels do not have a prognostic significance in the case of MNTI. The management options of MNTI include simple excision, complete enucleation with or without adjuvant treatments such as curettage and peripheral ostectomy, resection of the tumor either marginal or segmental, chemotherapy, radiotherapy, or combined treatment modalities. Two stems of thoughts arise in the case of the management of MNTI: resection versus conservative management. The extent of the lesions, effect on adjacent vital structures such as orbit, nasal cavity, and infratemporal area, age at diagnosis, margins of the excised specimen, and the number of recurrences dictate the treatment option to be opted for.

Enucleation is much preferable in the case of easily accessible and well-encapsulated cases of MNTI. Curettage or peripheral osteotomy benefits in the removal of microscopic infiltration of MNTI into surrounding bone. In the case of a tumor with macroscopic infiltration into adjacent structures, posterior lesions in which complete excision with clear margins is not possible, and in case of multiple recurrences, resection of the tumor is advisable. Resection of the entire tumor with a 5-mm safe margin is considered the standard treatment option by most surgeons [14]. But resection of the jaw in infancy or young individuals causes permanent functional and cosmetic deficit. Subtotal resection preserving the vital structures and maintaining anatomical barriers such as periosteum or orbital fat are advisable in cases where macroscopic margins are clinically normal. There are instances of spontaneous resolution even in case of residual tumor [15]. Carnevale and Mortelliti proposed the use of an operating microscope while treating MNTI to remove unseen remnants of the tumor to prevent recurrence [16]. Chrcanovic and Gomez concluded that enucleation with or without complementary treatment (curettage or peripheral osteotomy) would appear to be the most indicated therapy as curettage and resection have a high incidence of recurrence and morbidity, respectively [17]. Chemotherapy as a sole treatment option for MNTI was mentioned only in a few case reports. The mainstay of chemotherapy in the management of MNTI is as neoadjuvant chemotherapy if the tumor is inoperable [18] or when metastatic disease is present and as adjuvant chemotherapy in the case of disease progression even after two surgical interventions or showing signs of malignancy. Current Children's Cancer and Leukaemia Group (CCLG) guidelines indicate two regimens for MNTI, with the first involving cyclophosphamide and vincristine for benign but recurrent tumors. The second regimen of OPEC/ OJEC (vincristine (O), cisplatin (P), etoposide (E), cyclophosphamide (C), and carboplatin (J)) is reserved for persistently progressing tumors or malignant variants [19].

Radiotherapy is seldom used as a main treatment option for MNTI. It is indicated as an adjuvant option along with chemotherapy in a few cases of malignant and metastatic MNTI with no significant outcomes. The recurrence rate of MNTI ranges from 15% to 27% [20]. According to the latest systematic review of MNTI of jaw bones, the recurrence rate is 11.3%, and in our review, it is 15.2%. Reasons stated for recurrence are incomplete tumor excision, multifocal tumor, or tumor seeding during surgery [20]. According to Pontes et al., higher recurrence rates are seen in patients younger than 2 months old, who presented with distant metastasis or a treatment approach consisting of complete excision and radical surgery without margin clearance [1]. Rachidi et al. concluded that most recurrences occur within the first six months post-treatment and that cases diagnosed within the first two months of life have a high recurrence rate, which is similar to our study [5].

Even though a benign disease, it was mentioned that MNTI has malignant potential of 6.97%, which is higher compared to our study, which shows 2%. Metastasis to the cervical lymph nodes can be seen in malignant variants. Overall metastatic potential and distant metastasis of jaw MNTI are less compared to MNTI of the skull and brain. Combination therapy with surgery or chemotherapy with or without radiotherapy remains the treatment option for MNTI but with high recurrence rates. Regular follow-up is a requisite during the initial months of surgery or other forms of management as MNTI has a high incidence of recurrence during the initial months itself, with a mean recurrence time of 3.26 months and a maximum of 20 months, and 92% of recurrences are within four months. We advise a weekly follow-up during the first month, monthly follow-up for four months, and a regular follow-up for two years to rule out recurrence.

## Conclusions

According to the results of our analysis, we can conclude that MNTI is a benign infantile tumor of the jaw, mostly affecting the anterior maxilla, showing male predominance, which can be successfully managed with surgical intervention such as enucleation with or without complementary treatment. Chemoradiotherapy is indicated in cases of malignancies, which is rare, and in cases of recurrence, which cannot be managed with further surgical intervention. A minimum follow-up of up to four months upto two years is advisable to rule out recurrence.
